# Analysis of Microbial Colonization, Including Multidrug-Resistant Gram-Negative Bacteria, in High-Risk Pregnant Women

**DOI:** 10.1177/26884844251398715

**Published:** 2026-01-29

**Authors:** Anh Le, Maria Emilia Solano, Maurice Kappelmeyer, Anton Ivanov, Angela Koeninger, Edith Reuschel

**Affiliations:** 1 University Department of Obstetrics and Gynecology, Clinic St. Hedwig of The Order of St. John, University of Regensburg, Regensburg, Germany.; 2 Specialized dermatology practice, Burghausen, Germany.; 3 Laboratory of Translational Perinatology, University of Regensburg, Regensburg, Germany.; 4 nTwig Consulting GmbH, Kemnath, Germany.

**Keywords:** preterm deliveries, risk pregnancies, microbial colonization, multi-resistant gram-negative bacteria, perianal/vaginal swabs

## Abstract

**Introduction:**

Preterm birth (PTB) remains a global challenge in obstetrics and is a leading cause of neonatal morbidity. It is estimated that at least 40% of all PTB are due to intrauterine infections. Given the concerning trend of multidrug resistance and limited resources, especially in gram-negative bacteria, early detection of colonization and risk factors is essential to prevent adverse outcomes. In this study, we analyzed the associations between maternal microbial colonization in pregnancy, particularly considering the prevalence of multidrug-resistant gram-negative (MRGN) and its impact on prenatal pregnancy-related complications.

**Methods:**

This retrospective, single-center cohort study consisted of 596 high-risk pregnant women, who were admitted to the hospital between February 2016 and April 2018. Based on their pregnancy complications, which led to admission, three subgroups were considered: non-infection-related (Non-Infected), infection-related (Infected), and Cervical-Insufficient. A total of 1319 swab samples collected from skin, perianal area, and vagina were screened on culture media and were categorized as nonpathogenic, MRGN, pathogenic gram-negative, pathogenic gram-positive bacteria, and unicellular organisms, for example, yeast. Statistical analyses included chi-square test. *p*-Values <0.05 were considered significant.

**Results:**

The main findings of this study indicate a low prevalence of MRGN with 2% in each subgroup and an increased occurrence of pathogenic gram-negative bacteria in the perianal area (*p* < 0.05). However, no statistically significant association was found between microbial colonization and prenatal pregnancy-related complications. A combined screening of vaginal and perianal enhances the detection of pathogenic microorganisms, but no significant differences in pregnancy-related complications at admission were found. Additional perianal screening does not provide more evidence regarding the women’s health status.

**Conclusion:**

Therefore, general screening, including perianal swabs, cannot generally be recommended. Further research is needed to identify additional risk factors for the development of adverse pregnancy and neonatal complications.

## Introduction

Preterm birth (PTB) remains a global challenge in obstetrics and is a leading cause of neonatal morbidity, including neurological deficits, respiratory distress, and gastrointestinal complications.^1,2^ It is also associated with significant mortality, accounting for more than one million deaths in children under the age of 5 years annually.^3,4^ Worldwide, 15 million children are born prematurely as a result of preterm labor (PTL), which represents, for example, 10% of all births in the United States.^5,6^ In Europe, according to the *European Perinatal Health Report*, the preterm delivery rate was approximately 7.3%.^7^ PTB is defined as birth before 37 gestational weeks and can result from medically indicated deliveries, due to maternal or fetal genetics, or from various pathological processes. Spontaneous PTL and preterm premature rupture of membrane (PPROM) account for about 70% of PTBs. Main risk factors are vaginal infections, vascular disorders, disruptions of the maternal-fetal unit, as well as chronic stress and malformations of the fetus and/or uterus.^8^ It is estimated that at least 40% of all PTBs are caused by intrauterine infections.^9^ During pregnancy, there are multiple pathways leading maternal infections to the unborn fetus. Microorganisms may gain access through ascension from the vagina, *via* hematogenous dissemination, or after invasive procedures.^10^ It is thought that intrauterine infections lead to an increase in pro-inflammatory cytokines (interleukin [IL]-8, IL-1β, tumor necrosis factor-α), which stimulate the release of further inflammatory mediators associated with chorioamnionitis, prostaglandins, and matrix metalloproteinases, resulting in maternal uterine contractions and rupture of fetal membranes.^11^

The composition and diversity of the vaginal microbial colonization are potential risk factors for PTB and play an important role in its pathophysiology.^12,13^ In healthy women, the vaginal microbiome is typically dominated by Lactobacillus spp., including common species like *Lactobacillus gasseri*, *Lactobacillus crispatus*, *Lactobacillus jensenii*, and *Lactobacillus iners*. Of note, the role of *Lactobacillus iners* species in vaginal health is still unclear, since it can be detected in normal conditions as well as during vaginal dysbiosis, such as bacterial vaginosis, and therefore might also present pathological features.^14^ Changes in the vaginal microbiome that lead to an overcolonization of pathogens result in bacterial imbalance. Notable pathogens involved in this process include *Chlamydia trachomatis*, *Trichomonas vaginalis*, Group B Streptococci, *Gardnerella vaginalis*, as well as gram-negative bacteria such as *Klebsiella pneumoniae*, *Escherichia coli*), Enterobacter spp., *Pseudomonas aeruginosa*, and *Acinetobacter baumannii*. Pathogenic bacteria that colonize gestational tissues or the lower genital tract can be transmitted to the neonate.^15^ While Group B Streptococcus (*Streptococcus agalactiae*) remains the most common pathogen causing early-onset sepsis, pneumonia, and meningitis, gram-negative bacteria of nosocomial origin are often responsible for other severe infections and have been reported to be an emerging problem in neonatal care units.^16–18^ Furthermore, there have been relevant increases in antimicrobial resistance over the last few decades. Especially maternal colonization with extended-spectrum beta-lactamase (ESBL) *E. coli* is a significant risk factor for PTB and PPROM as well as neonatal infection.^19–21^ A previous study about the distribution of gram-negative bacteria in vaginal and cervical swabs from 239 pregnant women with PPROM or PTL yielded the following results: *E. coli* (90%), Klebsiella spp. (12%), Proteus spp. (9%), Pseudomonas spp. (5%), *Morganella morganii* (2%), Enterobacter spp. (2%), Citrobacter spp. (2%), and *Acinetobacter* (2%).^22^ Similar observations have been made in other studies with *E. coli* and Klebsiella being the most frequently isolated pathogenic species.^23^ Given the concerning trend of multidrug resistance and limited resources in obstetrics and neonatology, early detection of colonization and risk factors is essential to prevent adverse outcomes. In the present work, we aimed to analyze maternal microbial colonization during pregnancy, particularly with regard to the prevalence of multidrug-resistant gram-negative (MRGN) bacteria and their impact on prenatal pregnancy-related complications.

## Methods

The objective of this study was to investigate the associations between maternal microbial colonization, its location and classification in pregnancy, and its impact on pregnancy-related complications that led to admission. In addition, the prevalence of MRGN bacteria was determined.

### Study design, population, and ethical consideration

This retrospective, single-center cohort study was conducted at the Tertiary Maternity Clinic St. Hedwig of The Order of St. John, University Department of Obstetrics and Gynecology, University of Regensburg in accordance with the World Medical Association Declaration of Helsinki, which has been approved by the Ethical Commission of the University of Regensburg (reference: 24-3801-104).

Informed consent was obtained from all study participants. The examinations were performed in accordance with relevant institutional and national guidelines, regulations, and approvals. The study population consisted of 596 high-risk pregnant women with threatening PTB, but without PPROM and admitted to the hospital between February 2016 and April 2018. Maternal clinical characteristics were collected from their patients’ records. Women were designated “high-risk” not only because of threatened PTB but also based on the presence of comorbidities such as obesity, gestational diabetes, hypertension, or hypothyroidism, which increased their risk profile for pregnancy-related complications.

Swabs for specimen collection were taken from multiple sites, namely precisely from the lower third of the vagina, precisely from the perianal skin (not from the rectum), and/or from the inguinal skin. Also, in some cases, placental swabs were additionally obtained. To avoid cross-contamination of the obtained samples from each predetermined site on each study patient, separate swabs were used for each sample site. In cases where multiple swabs per patient were obtained (*e.g.*, from inguinal skin, vaginal and perianal sites), only the first swab per site was used for comparative statistical analysis.

Since follow-up and delivery data were not consistently available as some women delivered outside our center, analysis was restricted to pregnancy-related complications at the time of hospital admission.

Based on those complications, we divided the study population into four groups: non-infection-related (Non-Infected), infection-related (Infected), women with a shortened cervix uteri without clinical signs of an infection (Cervical-Insufficient), and not assignable. Infection was defined by high temperature, increased C-reactive protein, or leukocytosis. Non-infected mothers were those who were admitted due to complications not related to infection. The term “Not assignable” was used when the reason for admission could not be classified into any of the three groups described above.

### Microbiological analysis

After having collected the abovementioned samples of pregnant women, they were transported safely within a maximum of 2-hour time limit between collection and delivery to the nearby laboratory. Swab samples were streaked out on different culture media: MacConkey (MCK), Columbia agar with 5% sheep blood (COS), Enterococcus selective media with colistin and nalidixic acid (CNA), Gardnerella selective media (Gardnerella) (Co. bioMerieux), ESBL selective media, and vancomycin-resistant enterococcus (VRE) selective media (Co. Mast Diagnostik). The transfer was carried out under a laminar air hood. After an incubation period of 24 hours (MCK, COS, CNA), 48 hours (VRE), and 72 hours (Gardnerella) at 36°C, the microorganisms were identified through mass spectrometry (Vitek MS, Co. bioMerieux). Resistances were determined by EUCAST (Vitek 2, Co. bioMerieux).

MRGN bacteria are defined by the National German Commission of Hospital Hygiene and Infection Prevention (KRINKO) as resistant to commonly used antibiotics (extended-spectrum cephalosporins, carbapenems, quinolones, acylureidopenicillins). Severity of resistance is determined by multidrug resistance to more antibiotic categories, for example, three antibiotic categories (multidrug resistance to three or more antibiotic categories [3MRGN]).

The microbiological results of the collected swabs were categorized into one of five pathogenic groups, including nonpathogenic bacteria (gram-positive/negative nonpathogenic), MRGN bacteria (gram-negative multidrug resistant), pathogenic gram-negative bacteria (gram-negative pathogenic including Acinetobacter spp., Citrobacter spp., Enterobacter spp., *E. coli*, *K. pneumoniae*, *P. aeruginosa*, *Serratia marcescens*), pathogenic gram-positive bacteria (gram-positive pathogenic including Group B Streptococcus, *Staphylococcus aureus*, Enterococcus spp.), and other pathogens (unicellular organisms, *i.e.*, yeast, *G. vaginalis*, Candida spp.).

### Statistical analysis

In the descriptive analysis, numbers, percentages, median, and interquartile range (IQR) were calculated. Statistical analyses included a chi-square test. *p*-Values <0.05 were considered significant. All analyses were performed using R, Python, and the libraries Pandas, Sklearn, SciPy, NumPy, and Patsy.

## Results

### Sample collection and characteristics

**FIG. 1.**
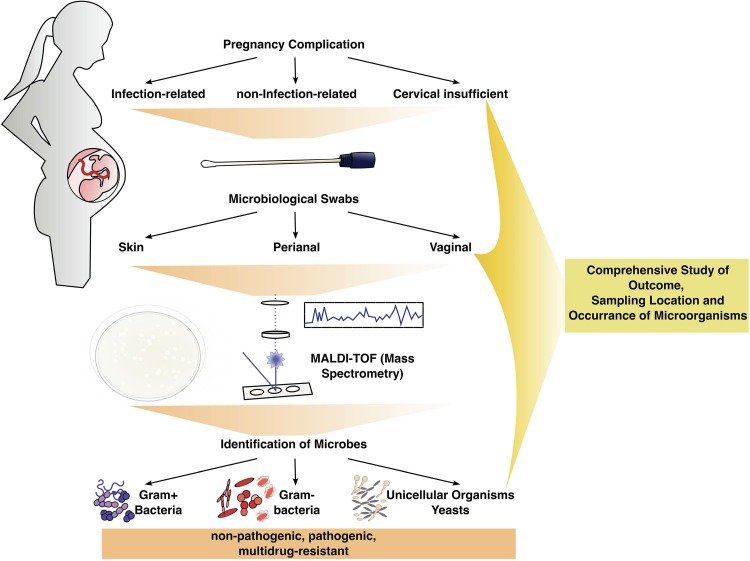
Pictogram flow of microbiological screening of high-risk pregnant women.

Between February 2016 and April 2018, a total number of 1699 swabs were collected from 596 high-risk pregnant women, aged between 17 and 48 years. Figure 1 illustrates the screening process used to obtain microbiological data, as well as sample identification and classification (Fig. 1). The swabs were taken precisely from inguinal and perianal skin, from the lower third of the vagina, and the placenta. Placental and follow-up swabs were excluded because of low numbers, redundancy, and unusability. Likewise, swabs from women who were not categorized as having infection-related or non-infection-related conditions and cervical insufficiency patients were not analyzed. The final number of swabs was 1319. The clinical characteristics of our study population are shown in Table 1. The number of participants varies due to a lack of information regarding characteristics and/or deliveries abroad (missing maternal and neonatal data of these patients). Thus, a part of the pregnant women underwent screening sampling during their hospitalization for threatened labor, but delivered finally outside our medical center. Therefore, out of 596 participants, the birth mode of only 480 pregnant women could be recorded, and just 479 live births were documented. Our study population included 154 (25.8%) women aged ≥35 years. At admission, 58.7% of the women were nulliparous, 25.8% were primiparous, and 15.4% were multiparous. Most of the study participants were recruited in their third trimester (65.1%), and approximately one-third (34.1%) of them were in the second trimester of pregnancy. Only three (0.5%) women were in the first trimester at the time of study inclusion. A risk profile consisting of inpatient antibiotic use (17.9%), obesity (29.0%), gestational diabetes (13.1%), hypothyroidism (12.2%), and hypertension (2.0%) was established, and the prevalence within our study population was investigated. The median of gestational weeks at delivery was 37 (IQR = 33–40). Among these births, cesarean sections accounted for 43.3% and vacuum extraction for 4.2% of the cases. Of the babies, 52.5% were delivered spontaneously, resulting in 97.9% live births (Table 1).

**Table 1.** Clinical Data of the Study Population

Parameter	No. of participants	Prevalence
Age (years) ≥35, *N* (%)	596	154 (25.8)
Parity	596	
Nullipara, *N* (%)		350 (58.7)
Primipara, *N* (%)		154 (25.8)
Multipara, *N* (%)		92 (15.4)
Multiple pregnancy, *N* (%)	595	75 (12.6)
Trimester	591	
1. 1–12 weeks of pregnancy, *N* (%)		3 (0.5)
2. 13–28 weeks of pregnancy, *N* (%)		203 (34.3)
3. 29–40 weeks of pregnancy, *N* (%)		385 (65.1)
Antibiosis (after admission), *N* (%)	595	107 (17.9)
Obesity^a^, *N* (%)	592	172 (29.0)
Gestational diabetes^b^, *N* (%)	595	78 (13.1)
Hypothyroidism^c^, *N* (%)	595	73 (12.2)
Hypertension^c^, *N* (%)	595	12 (2.0)
Birth mode	480	
- cesarean section, *N* (%)		208 (43.3)
- vacuum extraction, *N* (%)		20 (4.2)
- spontaneous delivery, *N* (%)		252 (52.5)
Live birth, *N* (%)	479	469 (97.9)

a Body mass index ≥ 30.

b Included diabetic and insulin-dependent gestational diabetes.

c Defined by medical treatment: l-thyroxine (hypothyroidism), antihypertensive medication (hypertension).

### Overall sample distribution of pathogens

**FIG. 2.**
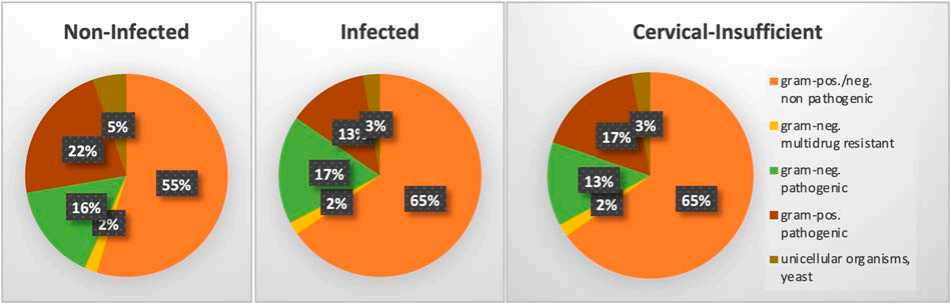
Overall distribution of maternal microbiological colonization from collected swabs (*N* = 1319) among Non-Infected (*N* = 148), Infected (*N* = 828), and Cervical-Insufficient (*N* = 343) study participants independent of the collection site. Microorganisms were categorized as follows: pathogenic (gram-positive/negative), nonpathogenic (gram-positive/negative), multidrug-resistant (gram-negative), pathogenic (gram-negative), pathogenic (gram-positive), and unicellular organisms, including yeast. Percentages of the total swaps are reported. Neg., negative; pos., positive.

Out of the 1319 swabs, 148 (11.2%) samples belonged to Non-Infected, 828 (62.8%) to the Infected, and 343 (26.0%) to the Cervical-Insufficient women. The classification into pathogenic groups and overall distribution among the Non-Infected group, the Infected group, and Cervical-Insufficient group are presented in Figure 2. Among all patients, we observed a higher prevalence of gram-positive and gram-negative nonpathogenic microorganisms followed either by gram-positive pathogenic or by gram-negative pathogenic bacteria, depending on the participant group. Unicellular organisms and yeast, and gram-negative multidrug-resistant bacteria accounted for a small proportion of the microorganisms in swabs from all patients (Fig. 2).

### Observed location-dependent distribution

**FIG. 3.**
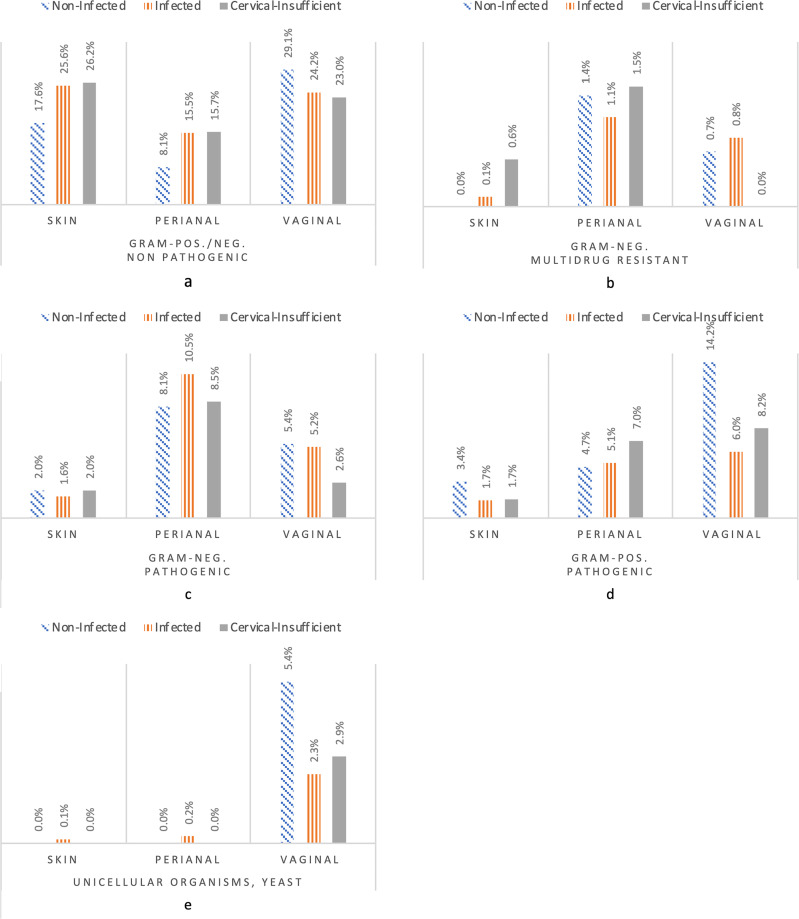
Percentage distribution of pathogenic groups: **(a)** gram-positive/negative nonpathogenic group, **(b)** gram-negative multidrug-resistant group, **(c)** gram-negative pathogenic group, **(d)** gram-positive pathogenic group, **(e)** unicellular organisms (yeast) among samples from different sites as inguinal skin, perianal skin, and vaginal specimen among the Non-Infected (*N* = 148), the Infected (*N* = 828), and the Cervical-Insufficient groups (*N* = 343).

To investigate a location-dependent distribution, we analyzed the microbial distribution across various sample sites (skin, perianal, and vaginal) within each microorganism subgroup. The colonization of the gram-positive/negative nonpathogenic group was higher on inguinal skin (Non-Infected group: 17.6%, Infected group: 25.6%, and Cervical-Insufficient group: 26.2%) and vaginal sites (29.1%, 24.2%, and 23.0%) compared with perianal sites (8.1%, 15.5%, and 15.7%) (Fig. 3a). Frequencies of gram-negative multidrug-resistant group were 1.4% (Non-Infected group), 1.1% (Infected group), and 1.5% (Cervical-Insufficient group) in perianal swabs versus 0.7%, 0.8%, and 0.0% in vaginal swabs (Fig. 3b). Gram-negative pathogenic bacteria were predominantly detected in perianal samples (Non-Infected group: 8.1%, Infected group: 10.5%, and Cervical-Insufficient group: 8.5%) compared with vaginal (5.4%, 5.2%, and 2.6%) and inguinal skin (2.0%, 1.6%, and 2.0%) specimens (Fig. 3c). In contrast, the presence of gram-positive pathogenic bacteria was observed more in vaginal swabs (Non-Infected group: 14.2%, Infected group: 6.0%, and Cervical-Insufficient: 8.2%) compared with perianal (4.7%, 5.1%, and 7.0%) and inguinal skin (3.4%, 1.7%, and 1.7%) swabs (Fig. 3d). Yeast and unicellular organisms were observed mainly in vaginal samples (Non-Infected group: 5.4%, Infected group: 2.3%, and Cervical-Insufficient group: 2.9%) rather than in inguinal and perianal skin samples (Fig. 3e). Derived from this observation, it was evident that relevant pathogenic microorganisms were mostly found in perianal and vaginal swabs (Fig. 3a–e).

Based on the observation that clinically relevant microorganisms were detected in vaginal and perianal swabs, we compared the prevalence of microorganisms in these locations for women with both swabs collected. This included 397 vaginal and perianal swabs from Non-Infected, Infected, and Cervical-Insufficient women (Table 2).

**Table 2.** Vaginal and Perianal Distribution of Gram-Positive/Negative Nonpathogenic, Gram-Negative Multidrug Resistant, Gram-Negative Pathogenic, Gram-Positive Pathogenic Bacteria, and Unicellular Organisms, Such as Yeast, Among the Non-Infected, the Infected, and the Cervical-Insufficient Groups (No. of Patients, Column-Wise %)

	Non-Infected (*N* = 32)				Infected (*N* = 256)				Cervical-Insufficient (*N* = 109)			
	Vaginal		Perianal		Vaginal		Perianal		Vaginal		Perianal	
	*n*	%	*n*	%	*n*	%	*n*	%	*n*	%	*n*	%
**Gram-pos./neg. nonpathogenic**	20	31.3	12	18.8	158	30.9	121	23.6	71	32.6	52	23.9
**Gram-neg. multidrug resistant**	1	1.6	2	3.1	6	1.2	9	1.8	0	0	5	2.3
**Gram-neg. pathogenic**	4	6.3	12	18.8	33	6.4	86	16.8	6	2.8	28	12.8
**Gram-pos. pathogenic**	6	9.4	6	9.4	42	8.2	39	7.6	23	10.6	24	11
**Unicellular organisms yeast**	1	1.6	0	0	17	3.3	1	0.2	9	4.1	0	0

Neg., negative; pos., positive.

#### Influence of vaginal and perianal sites on microbial colonization among maternal subgroups

Microbial colonization was compared between swab sites within each subgroup (Non-Infected, Infected, Cervical-Insufficient). In the Non-Infected group, no statistically significant differences were found in microbial distribution between perianal and vaginal swabs (χ^2^ = 7.33, df = 4, *p* = 0.119). In contrast, both the Infected group (χ^2^ = 43.45, df = 4, *p* < 0.001) and the Cervical-Insufficient group (χ^2^ = 31.19, df = 4, *p* < 0.001) showed significant differences in the microbial profiles by swab location. In these groups, gram-negative pathogenic bacteria were more frequently present in perianal swabs compared with vaginal ones, whereas unicellular organisms and yeast were more often found in vaginal swabs.

#### Microbial colonization in vaginal and perianal samples of mothers with diverse conditions

The analysis of microbial colonization in vaginal swabs revealed no statistically significant differences in the distribution of microbial groups across the maternal groups (Cervical-Insufficient, Infected, Non-Infected) (χ^2^ = 8.77, df = 8, *p* = 0.362). Similarly, microbial group distribution in perianal swabs did not differ significantly between the groups (χ^2^ = 5.75, df = 8, *p* = 0.676). These findings suggest that microbial group distribution at vaginal and perianal sites was independent of maternal pregnancy risk.

#### Impact of sampling site on the distribution of maternal subgroups within each microbial group

The association between microbial group and maternal subgroup was further analyzed separately for vaginal and perianal swabs. Again, no statistically significant association was observed. The microbial distribution across the Infected, Non-Infected, and Cervical-Insufficient study participants did not differ significantly in the vaginal swabs (χ^2^ = 8.77, df = 8*, p* = 0.362) nor in perianal swabs (χ^2^ = 5.75, df = 8, *p* = 0.676). These findings indicated that the presence of specific microbial groups did not significantly correlate with infection and cervical status, regardless of the sampling site.

#### Abundance of pathogenic microorganisms at vaginal and perianal sample sites

**FIG. 4.**
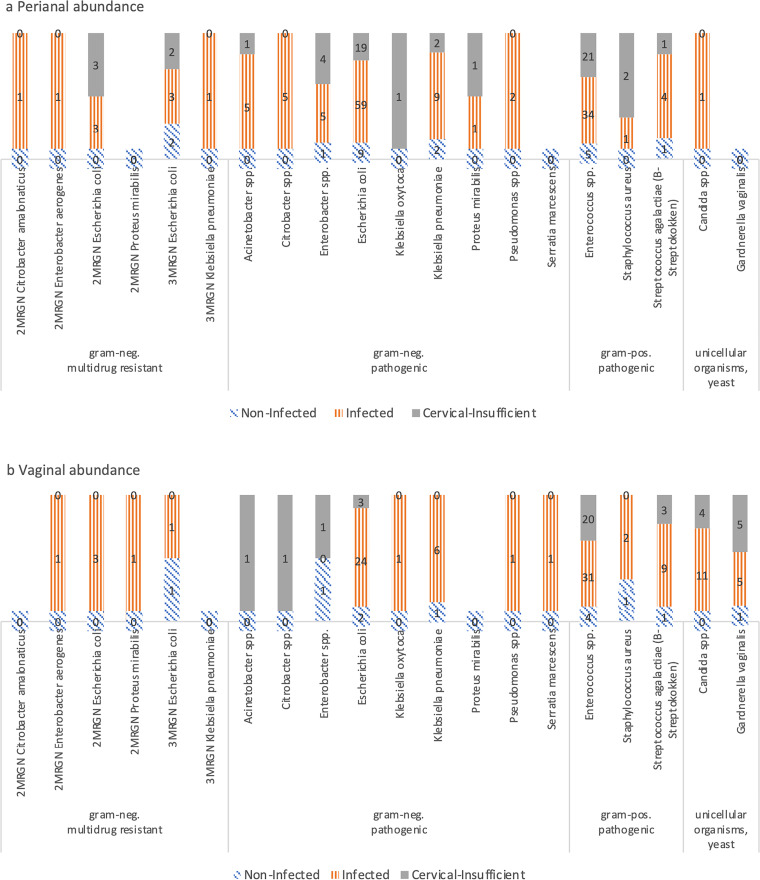
Microbial abundance of selected pathogens from **(a)** perianal and **(b)** vaginal samples among the Non-Infected (*N* = 64), the Infected (*N* = 512), and the Cervical-Insufficient (*N* = 218) groups.

Another approach involved a detailed analysis of the abundance of specific pathogenic bacteria, both gram-positive and gram-negative, in vaginal and perianal samples from women who underwent both types of swabs. The findings shown in Figure 4 revealed that *E. coli* and Enterococcus spp. were the most prevalent pathogens. They were followed by *K. pneumoniae* and *S. agalactiae*. Notably, among gram-negative multidrug-resistant bacteria, *E. coli* was also common, with both 2MRGN and 3MRGN variants observed. In vaginal samples, other 2MRGN bacteria were identified as *Enterobacter aerogenes* and *Proteus mirabilis*. In perianal samples, *K. pneumoniae* was identified as 3MRGN, whereas *Citrobacter amalonaticus* and *E. aerogenes* were identified as 2MRGN (Fig. 4).

## Discussion

While multidrug-resistant bacteria are a growing problem in health care, there is limited knowledge about their colonization in pregnant women. Nevertheless, acquiring these data is important for preventing fetal infections and potential long-term adverse outcomes. The overall prevalence of 2MRGN and 3MRGN in our study was 2%. 4MRGN and multi-resistant *S. aureus* (MRSA) were not at all detected. The QUARKS study, conducted between 2013 and 2015 in two German Obstetrics and Gynecology clinics, reported MRSA detection in pregnant women at 0.4% and ESBL at 2.6%, which was not higher than the general population’s prevalence.^24^ In addition, the screening of multidrug-resistant bacteria in high-risk pregnant women by Kunze et al. showed no higher colonization of MRSA and none of 4MRGN. However, the detection rate of 3MRGN was 8%.^25^ In contrast to these previous findings, in our study, the prevalence of multidrug-resistant bacteria like MRSA and MRGN was significantly low.

Most of the potentially pathogenic organisms detected in vaginal and perianal swabs support the results of former research.^24^ In our study, we observed that pathogenic Gram-negative bacteria were significantly more frequently detected in perianal swabs compared with vaginal swabs. Interestingly, this result was found in the Infected and Cervical-Insufficient groups. An explanation for this could be the natural occurrence of Enterobacteriaceae such as *E. coli* and *K*. *pneumoniae*, particularly in the intestinal tract. Warnke et al. investigated *E. coli*, *K. pneumoniae*, *P. aeruginosa*, and *A. baumannii* among various swab locations and found that intra-anal swabbing resulted in significantly higher bacterial quantities compared with perianal swabbing. In contrast, perianal sampling appeared to be more suitable than intra-anal sampling for detecting A*. baumannii.^26^* Given the fact that the resistance of gram-negative bacteria to common antibiotics is currently increasing, screening both, from vaginal and perianal areas, enhances the detection of relevant bacteria.

Examining the abundance of pathogenic microorganisms revealed that the most frequently isolated pathogenic gram-negative and gram-positive pathogens were *E. coli* and Enterococcus spp. Taking these findings from former studies into account, we were able to prove that lipopolysaccharides (LPS) of different gram-negative bacterial species induce distinct inflammatory responses and so exert different immunomodulatory functions. Especially, LPS of *E*. *coli* and *E*. *aerogenes* showed the strongest stimulatory activity by provoking the highest release of cytokines and chemokines.^27^ Furthermore, we were able to show that freshly isolated umbilical cord blood mononuclear cells responded specifically to *in vitro* stimulation by lysates of common vaginal gram-positive bacteria species, especially *Enterococcus faecalis*. This involved a substantial upregulation of the chemokines IL-8 and monocyte chemoattractant protein (MCP)-1, as well as macrophage inflammatory protein (MIP)-1α and MIP-1β by the bacterial lysates. *E. faecalis* presented even higher levels of MCP-1 secretion after stimulation compared with the other gram-positive bacteria lysates.^28^

In addition, these pathogens are found in aerobic vaginitis (AV). The association between AV and adverse pregnancy outcomes has been investigated in several studies.^29^ Moreover, a recent review indicates that even in the absence of symptoms, AV may present a risk factor for ascending chorioamnionitis and PROM.^30^ A meta-analysis by Chan et al. revealed growing evidence suggesting that, without clinical infection, the colonization of pathogenic microorganisms during pregnancy has the potential to impact the newborn. This assumption is supported by the results from Liu et al.^31^ They found that newborns from colonized mothers with *E. coli* showed neonatal morbidities.

It is well known that an abnormal maternal colonization is associated with adverse perinatal outcomes. A disruption of the maternal microbiota triggers an inflammatory immune response in the endometrium, leading to a disruption of placental development and defective placentation.^32–35^ Furthermore, disruption of the maternal microbiota can impact the development of the fetal microbiological colonization, which is involved in the pathogenesis of allergic diseases, autoimmune disorders, and even neurological deficits.

We aimed to investigate the impact of colonization with pathogenic microbes on prenatal pregnancy-related complications that led to admission. Although no statistically significant correlation was found between maternal colonization and pregnancy-related complications in this study, the high prevalence of potentially pathogenic organisms, especially gram-negative bacteria like *E. coli*, underscores the importance of monitoring microbial trends. The absence of a direct association suggests that colonization alone is insufficient to predict complications, pointing to the need for more integrative biomarkers that combine microbial, immunological, and clinical data.

Jain et al. discussed the pathways involved in chorioamnionitis in their review and pointed out that “sterile” intra-amniotic inflammation appeared to be more common as a causative agent for chorioamnionitis.^36^ This can be supported by the retrospective study of Oh et al., which determined the frequency of intra-amniotic infection and intra-amniotic inflammation in pregnant women diagnosed with clinical chorioamnionitis. They found that 66% of the patients had a negative amniotic fluid culture for microorganisms. In contrast, 24% of them had no evidence of either culture-proven intra-amniotic infection or inflammation.^37^ It becomes apparent that more research is needed to understand the interplay between colonization, inflammation, and infection that influences the pathogenesis of various prenatal pregnancy-related complications. By then, screening procedures and findings should be critically evaluated to limit false-positive results and overtreatment, which may lead to exhaust the current limited therapeutic approaches.^38^

As a retrospective study, our investigation has inherent limitations, including the absence of a predefined control group, incomplete follow-up in some cases, and the examination of neonatal outcomes. Some patients underwent screening, but delivered their babies outside our medical center, so we lack their follow-up and outcome data. We acknowledge that the use of antibiotics can impact infectious morbidity. The evaluation was, however, complicated due to the lack of antibiotic history outside our clinic and variable treatment of the pregnant women. We acknowledge that these constraints limit the generalizability of our findings. Future prospective studies with standardized follow-up protocols are needed to validate these results. Nevertheless, we postulate that this study provides valuable information regarding the impact of perinatal microbial colonization on the vulnerable group of high-risk pregnant women and contributes to the important goal of developing strategies to identify infection-triggered PTB as well as associated complications for both mother and child.

## Conclusion

The present study aimed to determine the location and classification of the maternal microbial colonization in high-risk pregnant women and the prevalence of MRGN bacteria. Interestingly, we could show that the overall rate of MRGN in our study was significantly low. Despite this result, we need to be aware of the rising problem of multidrug resistance, especially in gram-negative pathogens. However, a significant association with pregnancy complications was not found in this study. Although perianal swabs did not demonstrate a significant association with adverse outcomes, their inclusion enhanced the overall detection of gram-negative pathogens, which may be of importance in specific clinical contexts. Nonetheless, our findings suggest that routine perianal screening should be carefully evaluated to avoid unnecessary interventions. Further research and studies are required to better identify and categorize additional risk factors that can lead to adverse pregnancy and neonatal complications, and support the wise use of medical resources.

## Authors’ Contributions

A.L.: Project development, data collection/management, and article writing. M.K. and A.I.: Statistical analysis. M.E.S.: Discussion of results and editing of the final article. A.K.: Proofreading and final correction. E.R.: Project development, data collection/management, article writing, and editing.

## Abbreviations

3MRGN: multidrug resistance to three or more antibiotic categories
AV: aerobic vaginitis
CNA: colistin and nalidixic acid
ESBL: extended-spectrum beta-lactamase
IL: interleukin
IQR: interquartile range
LPS: lipopolysaccharides
MCK: MacConkey
MCP: monocyte chemoattractant protein
MIP: macrophage inflammatory protein
MRGN: multidrug-resistant gram-negative
MRSA: multi-resistant *Staphylococcus aureus*
neg.: negative
pos.: positive
PPROM: preterm premature rupture of membrane
PTL: preterm labor
PTB: preterm birth
spp.: species pluralis
VRE: vancomycin-resistant enterococcus
